# Design of a forward view antenna for prostate imaging at 7 T

**DOI:** 10.1002/nbm.3993

**Published:** 2018-07-18

**Authors:** Bart R. Steensma, Ingmar Voogt, Abe J. van der Werf, Cornelis A.T. van den Berg, Peter R. Luijten, Dennis W.J. Klomp, Alexander J.E. Raaijmakers

**Affiliations:** ^1^ University Medical Center Utrecht Utrecht the Netherlands

**Keywords:** 7T, prostate imaging, waveguide

## Abstract

**Purpose:**

To design a forward view antenna for prostate imaging at 7 T, which is placed between the legs of the subject in addition to a dipole array.

**Materials and methods:**

The forward view antenna is realized by placing a cross‐dipole antenna at the end of a small rectangular waveguide. Quadrature drive of the cross‐dipole can excite a circularly polarized wave propagating along the axial direction to and from the prostate region. Functioning of the forward view antenna is validated by comparing measurements and simulations. Antenna performance is evaluated by numerical simulations and measurements at 7 T.

**Results:**

Simulations of *B*_1_^+^ on a phantom are in good correspondence with measurements. Simulations on a human model indicate that the signal‐to‐noise ratio (SNR), specific absorption rate (SAR) efficiency and SAR increase when adding the forward view antenna to a previously published dipole array. The SNR increases by up to 18% when adding the forward view antenna as a receive antenna to an eight‐channel dipole array in vivo.

**Conclusions:**

A design for a forward view antenna is presented and evaluated. SNR improvements up to 18% are demonstrated when adding the forward view antenna to a dipole array.

**Abbreviations**
CoV: coefficient of variation
MRI: magnetic resonance imaging
MRS: magnetic resonance spectroscopy
PCB: printed circuit board
RF: radiofrequency
ROI: region of interest
SAR: specific absorption rate;
SAR_10g_: 10‐g averaged specific absorption rate
SNR: signal‐to‐noise ratio
T2w: *T*_2_‐weighted
TE: transverse electromagnetic
TSE: turbo spin echo
UHF‐MRI: ultrahigh‐field magnetic resonance imaging

## 1 INTRODUCTION

Diagnostic methods for prostate cancer can be improved by the development of techniques, such as high‐resolution magnetic resonance imaging (MRI), magnetic resonance spectroscopy (MRS) and diffusion‐weighted MRI.^1^, ^2^, ^3^, ^4^, ^5^, ^6^, ^7^, ^8^, ^9^, ^10^ These techniques can benefit from the gain in signal‐to‐noise‐ratio (SNR) that is provided by ultrahigh‐field MRI (UHF‐MRI, *B*_0_ ≥ 7 T). To date, multiple studies at 7 T have shown promising results for *T*_2_‐weighted (T2w) imaging^11^, ^12^ and ^1^H and ^31^P MRS^6^, ^13^, ^14^ on patients. However, UHF‐MRI is hindered by radiofrequency (RF)‐related limitations, such as interferences, rapid signal attenuation and high energy deposition.^15^, ^16^, ^17^, ^18^ These problems can be overcome using local transmit arrays with multiple transceive elements and RF phase shimming.^15^, ^19^, ^20^, ^21^, ^22^, ^23^, ^24^, ^25^, ^26^, ^27^, ^28^

In receive, optimal imaging performance is obtained using an endorectal coil.^1^, ^5^, ^25^, ^28^ The position inside the rectum provides a close vicinity to the prostate, resulting in very high SNR values. However, an endorectal coil suffers from an obvious and severe drawback in terms of patient comfort. As an alternative, reception can be performed with an external receive array which, for UHF‐MRI, may (partially) be a transceive array. Commonly used transceive surface array elements for prostate MRI are positioned around the pelvis in a belt‐like fashion. These elements emit and detect RF signals that propagate predominantly in the transverse plane to and from the prostate. As a result, the distance from the elements to the prostate is relatively large and the resulting SNR is much lower than with an endorectal coil. However, the prostate is located relatively close to the perineum (the region between the anus and the scrotum). Targeting the prostate from this region could provide a potential gain in SNR without invasive procedures. However, this approach is fundamentally different from the traditional approach in terms of signal polarization. The signal that is emitted or detected by this single antenna needs to have a circular polarization along the direction of propagation.

This work outlines the design of an external transmit receive element which is placed between the legs of a subject against the perineum. This element is able to transmit and receive a circularly polarized field in the longitudinal direction. It is therefore called the “forward view antenna”. The forward view antenna combines a cross‐dipole antenna with a small rectangular waveguide.^29^ Waveguides have been used in MRI previously in travelling wave imaging, where the bore of the MRI scanner is used as a waveguide.^30^, ^31^, ^32^ Dielectric waveguides have also been used as transmit elements for the imaging of the ankle and carotid arteries in the neck.^33^, ^34^ Closely resembling the use of a dielectric waveguide is the use of a cavity resonator enclosed by a layer of conductive material for use in nuclear magnetic resonance systems, which was described in a patent.^35^

In this study, a metallic water‐filled waveguide is used that guides a circularly polarized propagating wave along the longitudinal direction into the tissue, towards the prostate. The purpose of this study is to demonstrate the design and optimization of this antenna. Electromagnetic simulations are used to assess RF safety and the potential gain of using the forward view antenna. The forward view antenna is evaluated as a receive‐only element, in addition to an eight‐channel fractionated dipole array, in an in vivo setup on three volunteers.^21^

## 2 MATERIALS AND METHODS

### 2.1 Forward view antenna construction

**Figure 1:**
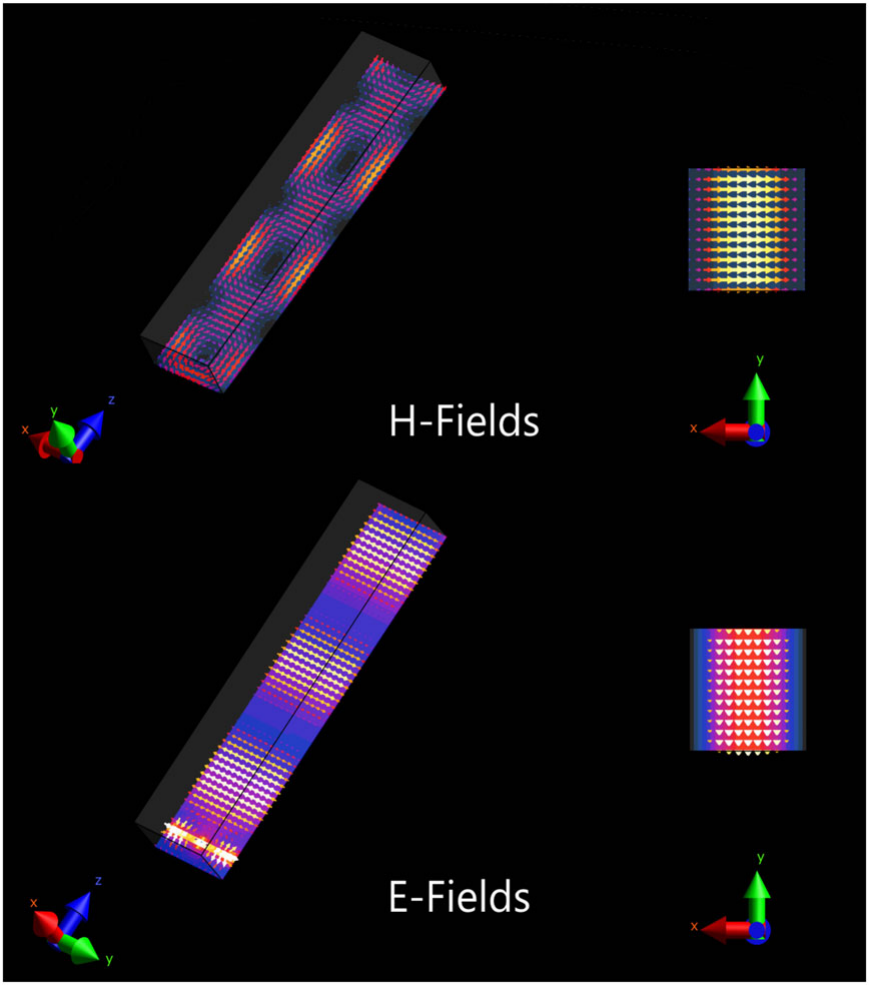
TE_01_ modes in a rectangular shielded waveguide. Both magnetic (top) and electrical (bottom) field components are shown. The TE_10_ mode has an identical field pattern, but is orthogonal to the TE_01_ mode. TE, transverse electromagnetic

The forward view antenna consists of a small rectangular copper‐shielded waveguide which is filled with deionized water and open at both ends. The orthogonal TE_01_ and TE_10_ modes (transverse electromagnetic modes) are excited in this waveguide.^36^ As these modes are orthogonal, their field patterns are equal, but the field lines point along orthogonal directions (in the yz and xz planes). The magnetic and electric field patterns for the TE_01_ mode are shown in Figure 1.

**Figure 2:**
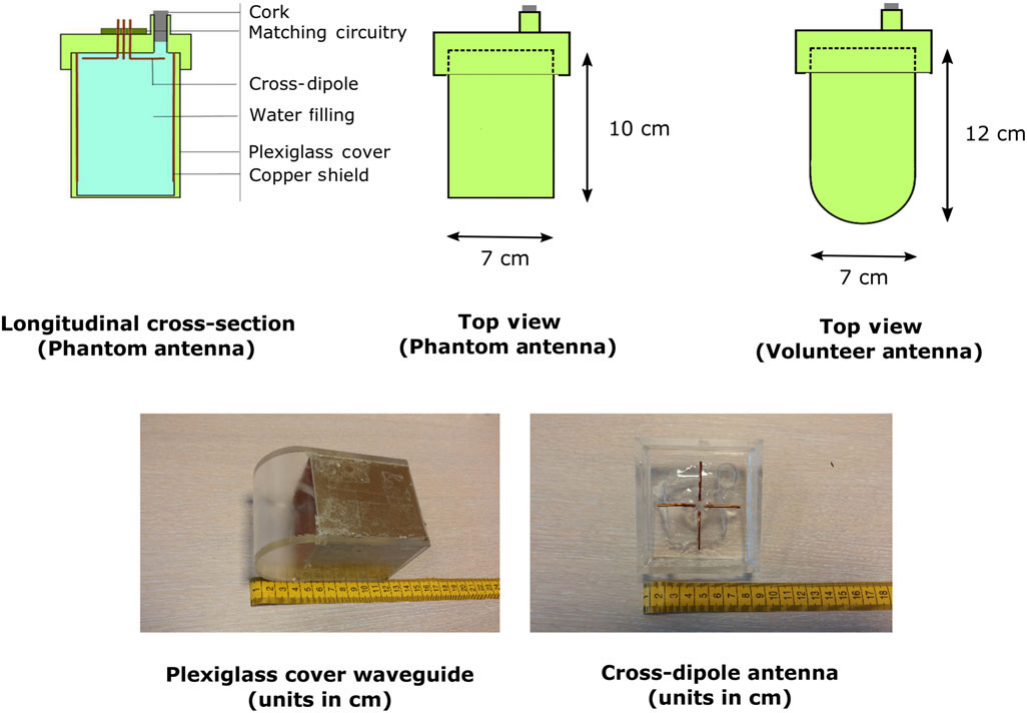
Schematic image of the forward view antenna. Different waveguide models have been used for the phantom experiments and the experiments on volunteers. Measurement units are in centimetres

The modes are excited by a small cross‐dipole antenna at the distal end of the waveguide. By feeding both separate dipole antennas with a 90° phase shift, a circularly polarized field is created by the antenna that propagates in the waveguide. This field can only propagate when the operating frequency (298.2 MHz at 7 T) is above the cut‐off frequency for the TE_01_ and TE_10_ modes in this specific waveguide structure. The cut‐off frequency of the lowest order TE modes of a rectangular conductive waveguide is given by the following equation^37^:

(1) $$f_{\mathrm{cut}-\mathrm{off},1,0}=\frac{c}{2a\sqrt{\varepsilon_{r}}}$$

where *c* is the speed of light, *ε*_*r*_ is the relative permittivity of the material inside the waveguide and *a* gives the inner width of the rectangular waveguide. Equation (1) shows that, by increasing the permittivity of the medium in the waveguide, the minimum dimensions of the waveguide can be decreased. The waveguide was filled with deionized water, which leads to a minimum waveguide width of 57 mm for a cut‐off frequency of 298.2 MHz and a relative permittivity of 78. The waveguide dimensions were therefore chosen to be 60 × 60 × 100 mm^3^ (slightly above cut‐off). A schematic image of the forward view antenna is shown in Figure 2.

**Figure 3:**
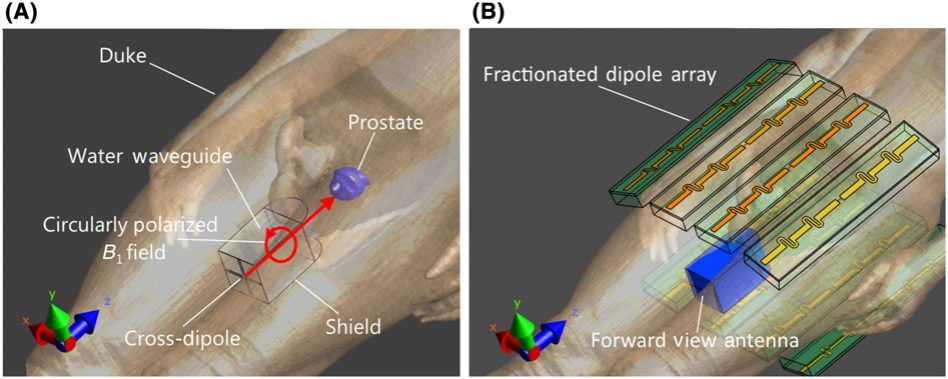
Schematic overview of the forward view antenna and the fractionated dipole antennas on the Duke model. A, Basic working principle of the forward view antenna. Red lines indicate the propagation direction and polarization of the transmit field. B, Imaging setup in which the forward view antenna is used in addition to the fractionated dipole array

The waveguide was constructed from a small Plexiglass box and four printed circuit board (PCB) patches were used as the conductive walls. The PCB patches were soldered together and covered in a layer of varnish to prevent oxidation of the copper. The plexiglass cover lies in between the body and the waveguide shield (plexiglass, 5 mm thick; 10 mm thick at the position of the dipole antennas). The cross‐dipole antenna was constructed from copper wire; the total length of a single antenna was 5 cm. Both dipoles were placed inside the waveguide and were connected to small copper patches outside of the waveguide, where the matching circuitry was placed. The tuning and matching network consisted of a parallel capacitor (8.2 pF) and a series capacitor (8.2 pF) for both channels. The maximum reflection coefficient for the forward view antenna was −12 dB. Coupling between the two orthogonal channels of the forward view antenna, as well as maximum coupling between the forward view antenna and fractionated dipole antennae, was low (−26 and − 23 dB). The geometrical design of the forward view antenna used for in vivo measurements deviated slightly from the initial rectangular design. The rectangular waveguide was extended with a half‐round end in order to maintain full contact with the tissue at the perineum. Both waveguide designs are presented in Figure 2. Figure 3 shows a schematic overview of the forward view antenna and its function as an addition to the fractionated dipole array.

### 2.2 Phantom simulations and scans

Initial simulations were carried out to optimize the length of the waveguide in terms of signal strength in a phantom, 6 cm away from the waveguide end. This distance was chosen because it equals the distance from the perineum to the prostate in the human model Duke (34 years old; body mass index, 22.4 kg/m^2^; height, 1.77 m; weight, 70.2 kg).^38^ The forward view antenna and a phantom with pelvic‐like electrical properties (250 × 250 × 200 mm^3^, *σ* = 0.4 S/m, *ε* = 34) were modelled in an electromagnetic simulation environment (Sim4Life, Zurich MedTech, Zurich, Switzerland).^39^ The length of the waveguide was varied, whilst the signal efficiency (*B*_1_^+^/√W) in the phantom was evaluated. MR experiments (MR system: Achieva 7 T, Philips Healthcare, Best, the Netherlands) were carried out on a phantom for a consistency check. A phantom was constructed from a small container filled with saline solution (380 × 240 × 150 mm^3^, 4 g/L, *ε* = 78, *σ* = 0.4 S/m). The forward view antenna was used as a transceive element, in which both ports of the forward view antenna were driven through a quadrature hybrid box (Philips Healthcare). *B*_1_^+^ maps were acquired using the actual flip angle method^40^ for a quantitative assessment of the efficiency, and confirmation that the simulation model matches the experimental results. Two *B*_1_^+^ maps were made at different input powers, because the *B*_1_^+^ values inside and outside the waveguide could not be mapped within the dynamic range of a single measurement. This experimental setup was modelled in an electromagnetic simulation environment (Sim4Life, Zurich MedTech) using the same electrical properties, and including the plexiglass structural parts, to verify the antenna concept.

### 2.3 Human model simulations

Electromagnetic simulations were carried out on human model Duke of the virtual family^38^ to evaluate the performance of the forward view antenna in addition to the fractionated dipole array setup.^21^ The following setups were evaluated: eight fractionated dipole antennas; the two‐channel forward view antenna; eight fractionated dipole antennas in combination with the two‐channel forward view antenna. The same simulation setup was used for the three experiments, in which the unused elements were still present, but not active. Tissue in the area of the legs that overlapped with the forward view antenna was cut away, in order to fit the forward view antenna between the legs of Duke.

The resulting field distributions were exported to Matlab. The electric fields were used to calculate 10‐g averaged quality matrices (Q‐matrices) and virtual observation points.^41^ Based on the *B*_1_^+^ distributions per channel and the virtual observation points, the average *B*_1_^+^ in the prostate, normalized by the square root of the maximum 10‐g averaged specific absorption rate (SAR_10g_; SAR efficiency), was optimized using the Matlab routine fmincon (Mathworks, Natick, MA, USA). The sum of the absolute values of the Q‐matrix was calculated to obtain the maximum possible peak SAR_10g_ value given unity input power for all channels and any phase setting.^42^ This value represents the maximum possible SAR value that can be obtained with phase‐only *B*_1_^+^ shimming. Receive performance was evaluated by combining the resulting *B*_1_^−^ fields, normalized by input current, with Roemer's sensitivity‐weighted corrected sum‐of‐squares method^43^ into relative SNR maps. A separate simulation was performed to compare the field distributions of the fractionated dipole array with and without the passive forward view antenna present. The results are shown as Supporting Information.

### 2.4 Volunteer scans

The forward view antenna was tested on three volunteers in receive‐only mode. These scans were conducted with the approval of our Institutional Review Board. Three healthy male volunteers (22, 26 and 36 years old; body mass index, 24.4, 20.9 and 26 kg/m^2^) were scanned using the forward view antenna and eight fractionated dipole antennas.^21^ Three different combinations of receive elements were reconstructed in post‐processing: receiving with the fractionated dipole antennas only, receiving with the forward view antenna only and receiving with all elements combined. In all cases, only the fractionated dipole array was used for transmission. Low flip angle gradient echo scans (Surveys) were acquired to obtain general insight into the performance of the different receive setups. To evaluate SNR in the prostate, the method of Kellman et al.^44^, ^45^ was used for the reconstruction of SNR scaled images. Raw data were exported for every separate receive channel; the different combinations of receive elements were all derived from the same dataset. Receive combination was performed using the sensitivity‐weighted sum‐of‐squares method.^43^ The following imaging parameters were used: three‐dimensional fast field echo sequence; field of view, 100 × 369 × 8 mm^3^; voxel size, 1.56 × 2.65 × 2 mm^3^; three slices; flip angle, 1°; echo time, 10 ms; repetition time, 50 ms. SNR was evaluated as the average signal in the prostate, considering only the middle slice of the SNR scaled images. In one example volunteer, a high‐resolution T2w image was acquired, and reconstructed with different combinations of receive setups (forward view antenna, fractionated dipole array, both) and SNR scaling. The following imaging parameters were used for the T2w acquisition: two‐dimensional spin echo sequence; field of view, 250 × 421 × 3 mm^3^; voxel size, 0.7 × 0.7 × 3 mm^3^; flip angle, 90°; flip angle refocusing pulse, 180°; echo time, 90 ms; repetition time, 5000 ms; multishot turbo spin echo (TSE), with TSE factor 17.

## 3 RESULTS

### 3.1 Phantom scans and simulations

**Figure 4:**
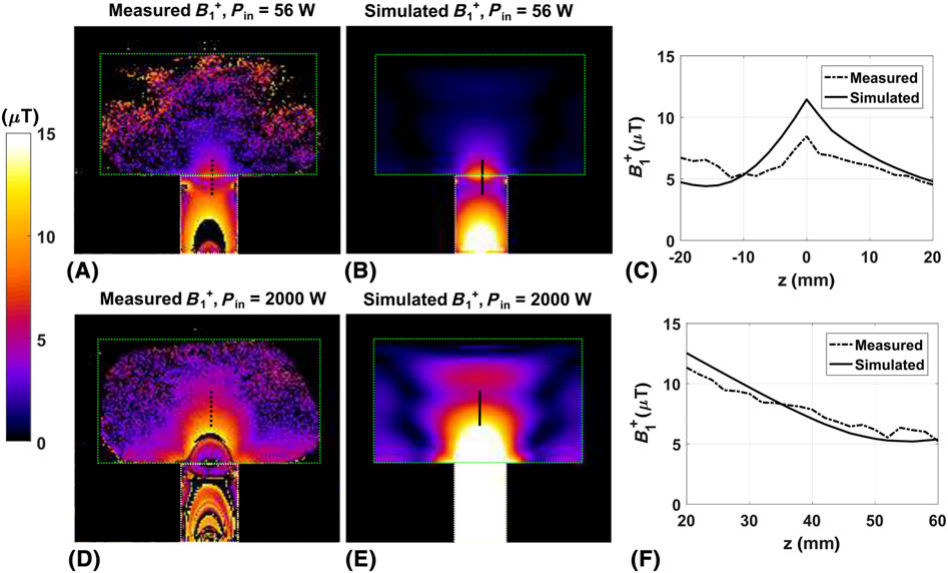
Measured (A) and simulated (B) transmit field of the forward view antenna, using an input power of 56 W. C, Simulated and measured transmit field along the black lines indicated in (A,B). D–F, The same results, but for an input power of 2000 W and for a different positioning of the black lines. The outlines of the waveguide and the phantom are indicated by the white and green lines in all figures. The transmit field was mapped using the actual flip angle (AFI) sequence and the following scan parameters: field of view, 320 × 320 × 30 mm^3^; voxel size, 2 × 2 × 10 mm^3^; echo time, 2 ms; repetition time, 50/250 ms; flip angle, 65°

The results of the optimization of length versus transmit efficiency are available as Supporting Information. It is shown that the length of the waveguide has no influence on the transmit efficiency in the phantom, which is to be expected from a lossless waveguide. The results of simulations and measurements on a phantom are shown in Figure 4. Figure 4A, B shows the measured and simulated *B*_1_^+^ maps for an input power of 56 W. Figure 4C shows a line plot through the measured and simulated data, as indicated by the black lines in the figures. Figure 4D–F shows the same data, but for an input power of 2000 W. The measured field pattern in the waveguide corresponds very well to the simulated pattern, which indicates that the waveguide works as expected. However, close to the interface between the element and the phantom, measured *B*_1_^+^ appears to be lower than expected from simulations. A slight mismatch in modelling the interface between the phantom, which consists of plexiglass and a thin layer of air, may have caused a deviation between simulations and measurements in this region of rapidly changing *B*_1_^+^.

### 3.2 Human model simulations

**Figure 5:**
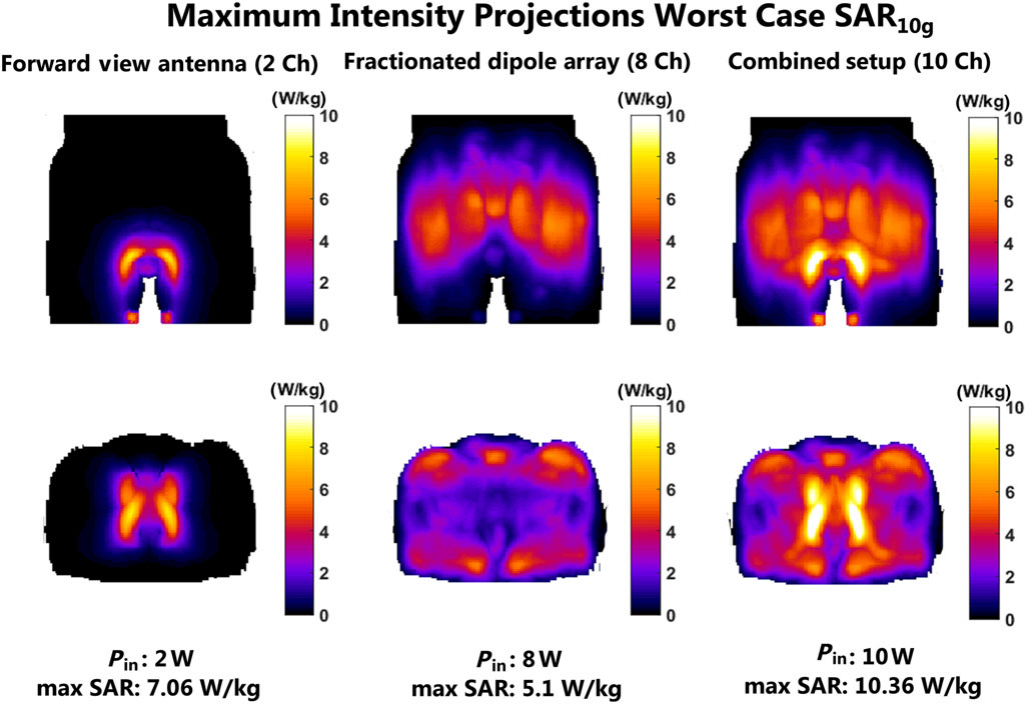
Worst‐case peak 10‐g averaged specific absorption rate (SAR_10g_) distributions for different combinations of the dipole antenna array and the forward view antenna on the human model Duke, given unity input power for each channel. Total input power is displayed below each simulation setup

Figure 5 shows maximum intensity projections of the worst‐case SAR_10g_ distributions, considering phase‐only shimming and equal input power for all transmit channels. The forward view antenna has an increased worst‐case local peak SAR (7.1 W/kg) relative to the fractionated dipole array (5.1 W/kg). The worst‐case local peak SAR for the fractionated dipole array in this simulation, where the forward view antenna is also present, is exactly the same as the worst‐case local peak SAR reported for the fractionated dipole array without passive forward view antenna present.^21^ The worst‐case peak SAR is highest when both the forward view antenna and the fractionated dipole array are used (10.4 W/kg). When the forward view antenna is used, the highest peak SAR values occur close to the proximal end of the waveguide; two SAR peaks can be seen at both sides of the waveguide. Constructive interference between the transmit fields of the fractionated dipole array and the forward view antenna causes a further increase in these SAR values when the combined setup is used.

**Figure 6:**
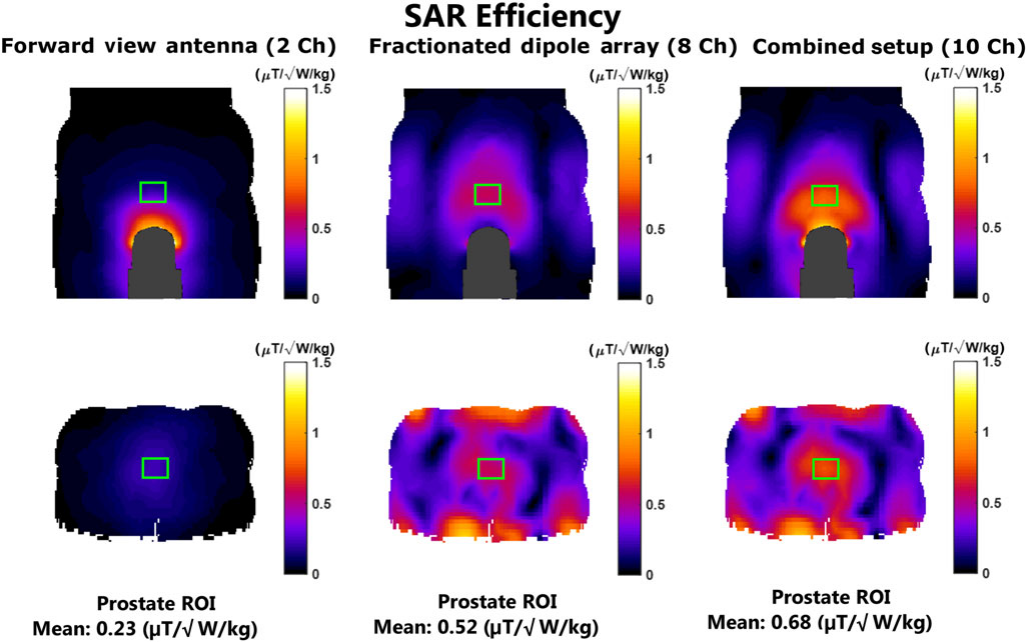
*B*_1_^+^ field distributions in the model Duke normalized to maximum local peak 10‐g averaged specific absorption rate (SAR_10g_; SAR efficiency) using the indicated antenna setups. The numbers next to each setup indicate the number of transmit channels. Phase and amplitude shimming was performed for every setup to generate SAR efficiency in the prostate region. ROI, region of interest

Figure 6 shows *B*_1_^+^ distributions normalized to peak SAR_10g_ (SAR efficiency) for an optimized drive phase and amplitude of each transmit channel. When using the forward view antenna alone, a SAR efficiency of 0.23 $\mathrm{\mu T}/\sqrt{W/\mathrm{kg}}$ is reached. With the fractionated dipole array, the SAR efficiency is 0.52 $\mathrm{\mu T}/\sqrt{W/\mathrm{kg}}$. With the combined setup, the SAR efficiency increases to 0.68 $\mathrm{\mu T}/\sqrt{W/\mathrm{kg}}$. This implies that using the forward view antenna and the fractionated dipole array can improve the SAR efficiency by 30.8%, compared with the use of the fractionated dipole array only.

**Figure 7:**
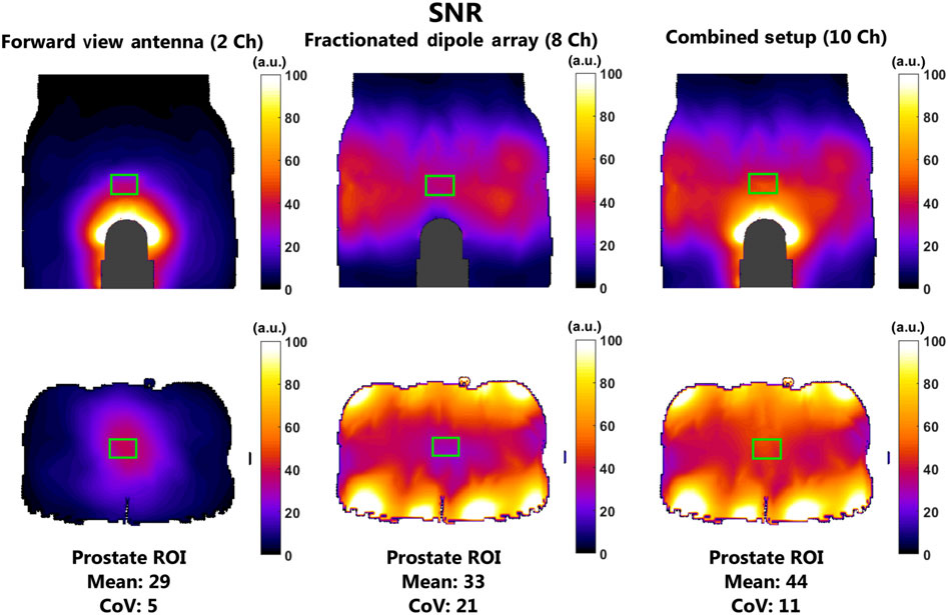
Signal‐to‐noise ratio (SNR) field distributions in the model Duke using the indicated antenna setups. SNR was obtained by combining sensitivity (*B*_1_^−^) maps with the sum of squares method. The results show that the forward view antenna potentially provides a 33% increase in SNR in comparison with the array of fractionated dipoles alone. CoV, coefficient of variation; ROI, region of interest

Figure 7 shows the simulated SNR maps for different receive setups. With the forward view antenna alone, an SNR of 29 is reached in the prostate, whereas the fractionated dipole antenna alone achieves an SNR of 33. Combining the fractionated dipole array and the forward view antenna leads to an SNR of 44, which is 33% higher than for the fractionated dipole array alone.

### 3.3 Volunteer scans

**Figure 8:**
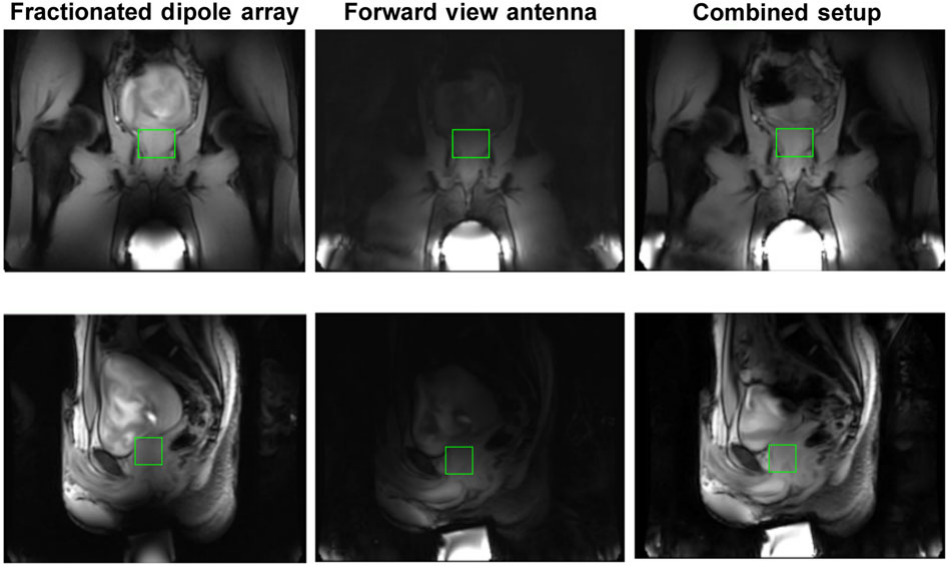
Survey scans acquired with different antenna combinations for receipt. The fractionated dipole array is used for transmission in all cases. The prostate is delineated by the green box. Imaging parameters: Multislice 2 dimensional (M2D) fast field echo sequence; field of view, 250 × 355 × 30 mm^3^; voxel size, 2 × 2 × 10 mm^3^; three slices; flip angle, 15°; turbo field echo (TFE) factor, 64; echo time, 4.93 ms; repetition time, 11 ms

Low flip angle images for three different setups are shown in Figure 8. When only the fractionated dipole antennas are used, the signal intensity close to the perineum is low. The two channels of the forward view antenna receive a signal that is highly intense close to the waveguide, but decays strongly when moving away from the waveguide. The signal intensity in the prostate region shows a strong gradient. Both observations also apply to the simulated receive fields in the prostate (Figure 7). The image of the combined setup looks comparable with that obtained with the fractionated dipole antennas only, although the signal void close to the perineum is no longer present.

**Figure 9:**
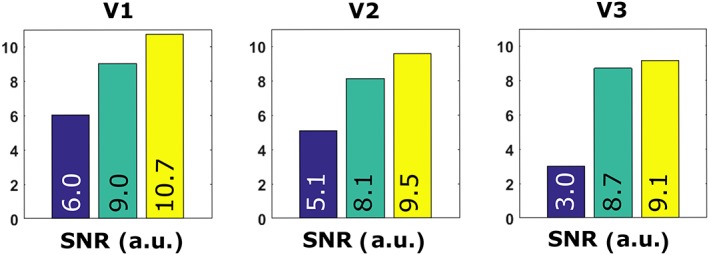
Signal‐to‐noise ratio (SNR) values obtained in a rectangular region of interest (ROI) inside the prostate for the three volunteers. SNR increases up to 18% when a forward view antenna is used in combination with the fractionated dipole array, as compared with the fractionated dipole array only

Figure 9 shows the average SNR in the prostate for the three volunteers. It is demonstrated in all volunteers that adding the forward view antenna as a receive element improves SNR compared with a situation in which only the fractionated dipole array is used. In volunteer 1, SNR increases from 9 to 10.7 (+19%), in volunteer 2 from 8.1 to 9.5 (+17%) and in volunteer 3 from 8.7 to 9.1 (+5%). The average increase in SNR over all volunteers and slices is 13.6%. The distance from the tip of the forward view antenna to the tissue was measured in survey images for all volunteers. This distance was 7.4, 7.9 and 12.55 cm for volunteers 1–3. Despite the round end of the forward view antenna, a small air gap is still present between the perineum and the forward view antenna for all volunteers.

**Figure 10:**
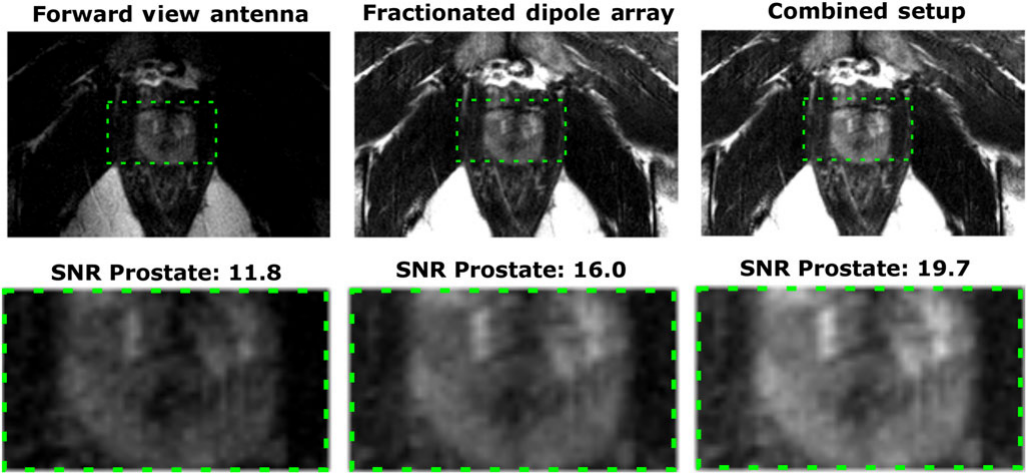
*T*_2_‐weighted (T2w) images for an example volunteer (V1), with signal‐to‐noise ratio (SNR) scaled reconstruction. The same transmit setup (eight fractionated dipole antennas) was used in all cases, but with different receive setups. The green region of interest (ROI) indicates a region in the prostate in which SNR was evaluated. The following scan parameters were used: field of view, 240 × 421 × 3 mm^3^; voxel size, 0.7 × 0.7 × 3 mm^3^; echo time, 90 ms; repetition time, 5000 ms; flip angle, 90°

Figure 10 shows a T2w image, reconstructed with different receive setups, and the average SNR values in an indicated region of interest (ROI) in the prostate. As expected, the highest SNR is obtained when both the forward view antenna and the fractionated dipole array are used in receive mode.

## 4 DISCUSSION

Simulations on a phantom show that the *B*_1_ field inside the forward view antenna has a circular polarization when using the right phase offset for both channels, the *B*_1_^+^ field in the waveguide is highly intense and the *B*_1_^−^ field is very low. A comparison of simulation and experimental results in a phantom shows that the simulated field patterns correspond to the experimental data. The correspondence in field patterns shows that the forward view antenna behaves as it is supposed to, i.e. it generates a circularly polarized wave in the longitudinal direction.

Simulations in a human model show that adding the forward view antenna to the fractionated dipole array can lead to an increase in SAR efficiency of 30.8%. However, the worst‐case SAR levels for this combined setup (10.4 W/kg) and for the forward view antenna (7.1 W/kg) are much higher than for the fractionated dipole array (5.1 W/kg). Simulated SNR maps show that the use of the combination of a fractionated dipole array and the two‐channel forward view antenna yields the highest SNR in the prostate in receive mode.

Although it is possible to increase SAR efficiency when adding the forward view antenna, the worst‐case SAR also increases strongly. As the worst‐case SAR is the current metric that is used at our site to set safe upper limits on average power, the forward view antenna was not used as a transmit element on volunteers in this work. The use of the forward view antenna in transmit mode on volunteers would require an update of the safety evaluation methods that are used at our site, including the use of multiple human models and MR thermometry for validation. This is ongoing work, and is considered to be beyond the scope of this article.^46^ For the volunteer experiments, the forward view antenna was evaluated for receive purposes only. SNR measurements in a volunteer demonstrate that a combination of the forward view antenna and the fractionated dipole array in receive mode leads to the best SNR. Adding the forward view antenna to the fractionated dipole array in receive can lead to an SNR increase of 19% in the prostate, but this is not the case for all volunteers. In the case of volunteer 3, SNR increases by only 5% when adding the forward view antenna to the fractionated dipole array. In this volunteer, the distance from the antenna to the tissue is relatively large (12.6 cm). This observation leads to the conclusion that anatomic differences and rigorous placement of the forward view antenna are important for its performance. In spite of the adapted shape of the waveguide end, the tissue and the waveguide are not fully connected, which results in reflection at the waveguide–subject interface.

The high signal intensity in the forward view antenna increases the dynamic range in the image. This can cause problems in the analog to digital converter (ADC) scaling of the image, and it can also harm the visibility of neighbouring anatomic structures. This issue could be resolved by replacing the deionized water in the forward view antenna by deuterium to remove the signal from the waveguide.^33^

Although this article describes the use of the forward view antenna for prostate imaging, several other targets could be imaged as well. For example, anatomical targets in the same area, such as the rectum, could benefit from the additional SNR provided by the forward view antenna. The brain could be a potential target for the forward view antenna, as the antenna could be used to realize improved coverage of the cranial side of the brain.

Endorectal coils provide another possibility to improve SNR and SAR efficiency for prostate imaging when adding them to an external transceiver array.^1^, ^5^, ^25^, ^28^ Reported SNR improvements when adding an endorectal coil to an external transceiver array range from 40% to 367%, which is an order of magnitude larger than the SNR improvement caused by adding the forward view antenna. However, the use of an endorectal coil can cause inhomogeneity in the signal, which is clearly visible in Figure 7d in the work of Ertürk et al.^25^ In addition, an endorectal coil causes patient discomfort.^47^

The limitations of the work described here mainly relate to the ergonomic design and the placement of the forward view antenna. Using a non‐shielded waveguide, which has no cut‐off frequency, or a waveguide composed of a high‐permittivity material would make it possible to design a smaller waveguide which is beneficial for ergonomics. These options have not been explored in this work, and could make it easier and more subject friendly to use the forward view antenna. The results presented in this work show that the SNR performance of the forward view antenna differs according to subject. Although a mechanical placeholder was made to fixate the forward view antenna, more effort should be applied to integrate the forward view antenna in the scanner bed. This would ensure full contact between the forward view antenna and the body, thus leading to maximum efficiency. The clinical T2w image shown in Figure 10 was acquired using a vendor‐available T2w sequence. Using the same scan parameters as in the work of Maas et al.^11^ could further improve the image quality, but would require the use of modified RF pulses and a modified sequence, which is considered to be beyond the scope of this work.

## 5 CONCLUSION

A forward view antenna was designed for prostate imaging at 7 T. The waveguide length of the antenna design was optimized in simulations. The forward view antenna functions as predicted in simulations on a phantom. By adding the forward view antenna to an array of fractionated dipole antennas, the SNR was improved as compared with the fractionated dipole array only.

## Supporting information


**Figure S1**: transmit efficiency in the phantom, 6 cm away from the round tip of the forward view antenna, as a function of waveguide length.
**Figure S2**: B_1_^+^‐fields and SAR distributions for a setup of 8 fractionated dipole antennas with and without the forward view antenna present as a passive element. All slices cut through the center of the prostate, which is marked blue in the center of the images.
