# Supplementary material for: Design of a forward view antenna for prostate imaging at 7 T
Source: NMR Biomed. 2018 Jul 18;31(9):e3993. doi: 10.1002/nbm.3993 (PMC6175442; doi:10.1002/nbm.3993)
Supplement: Supplementary file 1 — Figure S1: transmit efficiency in the phantom, 6 cm away from the round tip of the forward view antenna, as a function of waveguide length. Figure S2: B1 +‐fields and SAR distributions for a setup of 8 fractionated dipole antennas with and without the forward view antenna present as a passive element. All slices cut through the center of the prostate, which is marked blue in the center of the images. [file NBM-31-na-s001.docx]

**Supplementary material**


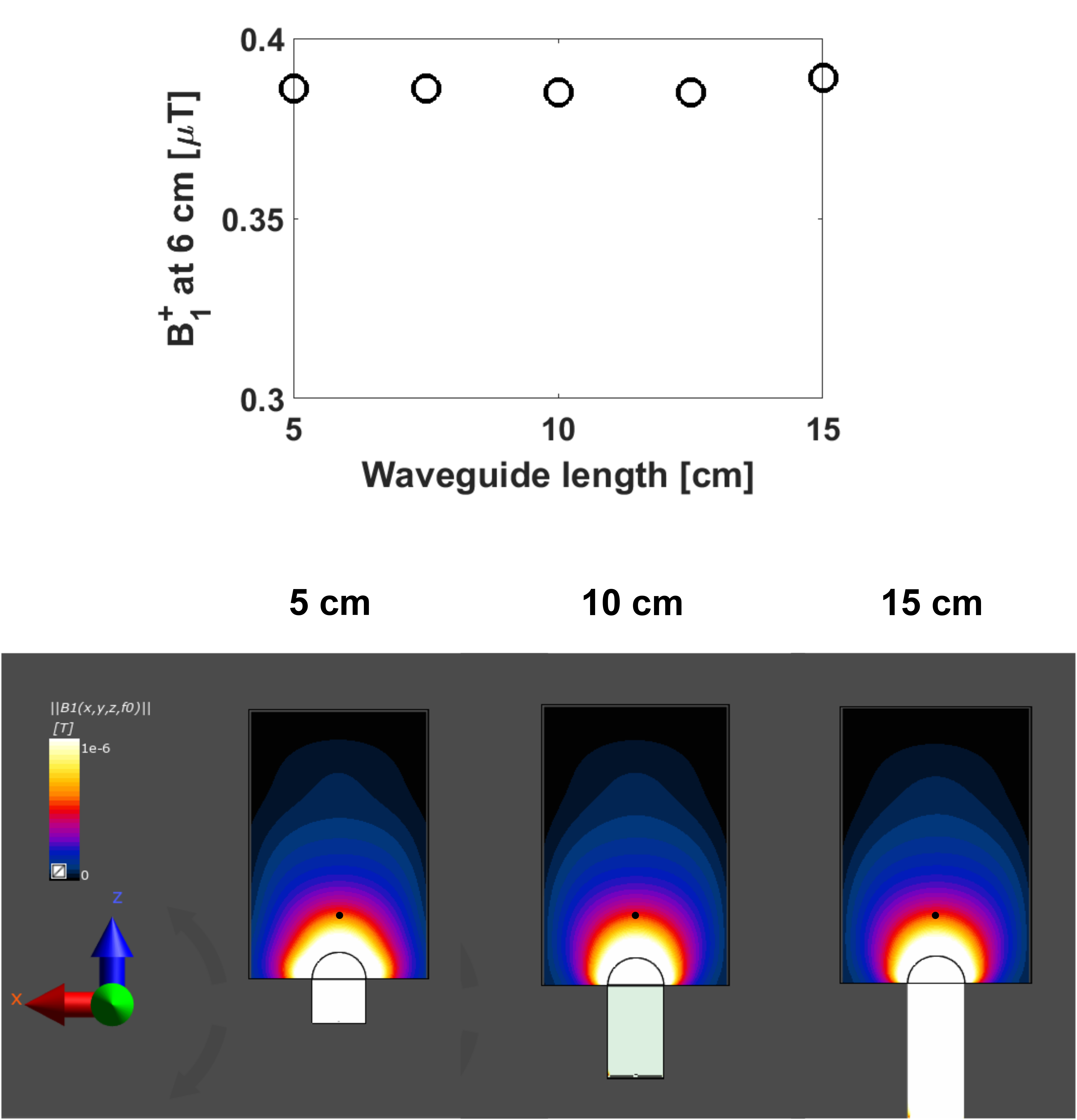


Figure S1: transmit efficiency in the phantom, 6 cm away from the round tip of the forward view antenna, as a function of waveguide length.


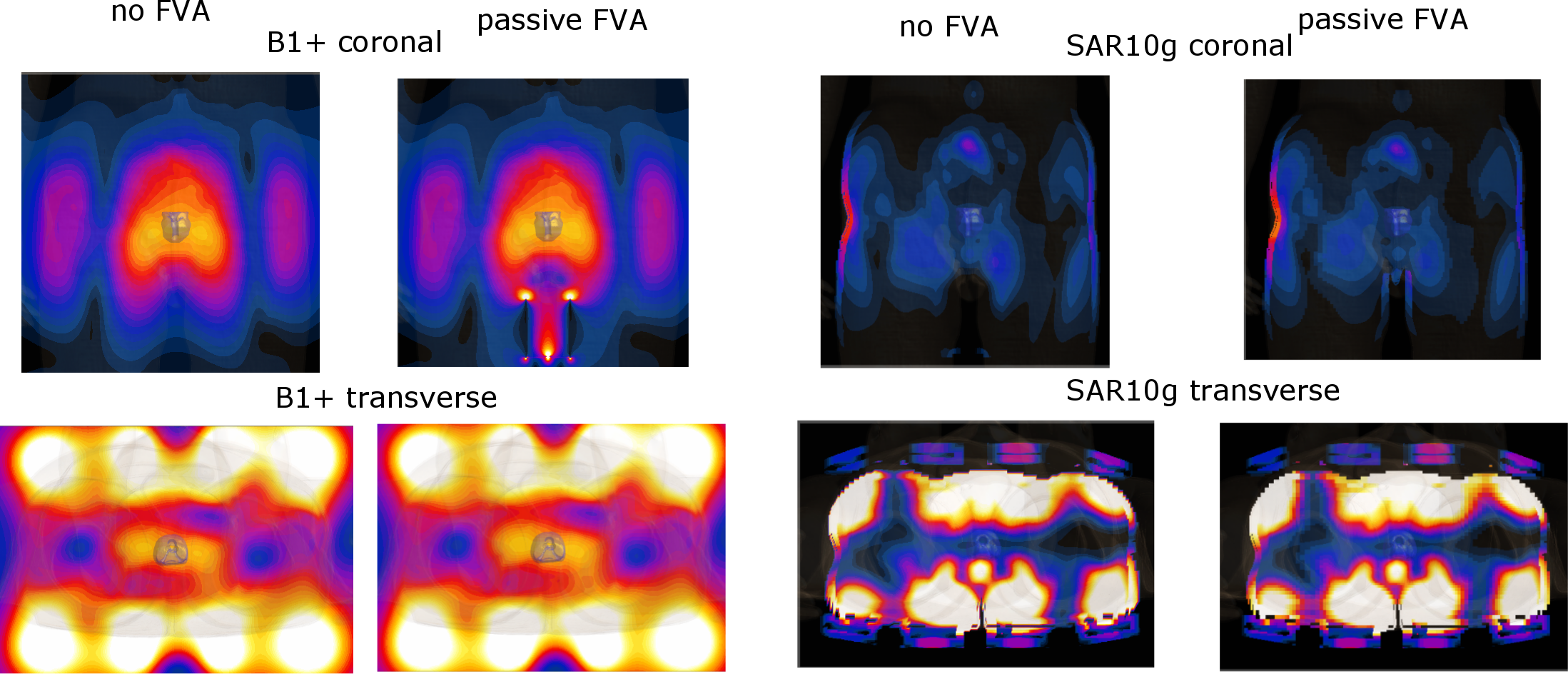


Figure S2: B_1_^+^-fields and SAR distributions for a setup of 8 fractionated dipole antennas with and without the forward view antenna present as a passive element. All slices cut through the center of the prostate, which is marked blue in the center of the images.
